# Synaptic Dysfunction in Multiple Sclerosis: A Red Thread from Inflammation to Network Disconnection

**DOI:** 10.3390/ijms22189753

**Published:** 2021-09-09

**Authors:** Laura Bellingacci, Andrea Mancini, Lorenzo Gaetani, Alessandro Tozzi, Lucilla Parnetti, Massimiliano Di Filippo

**Affiliations:** 1 Section of Neurology, Department of Medicine and Surgery, University of Perugia, 06132 Perugia, Italy; bellingacci@gmail.com (L.B.); mancini1andrea@gmail.com (A.M.); lorenzo.gaetani@unipg.it (L.G.); lucilla.parnetti@unipg.it (L.P.); 2 Section of Physiology and Biochemistry, Department of Medicine and Surgery, University of Perugia, 06132 Perugia, Italy; alessandro.tozzi@unipg.it

**Keywords:** multiple sclerosis, synaptopathy, inflammation

## Abstract

Multiple sclerosis (MS) has been clinically considered a chronic inflammatory disease of the white matter; however, in the last decade growing evidence supported an important role of gray matter pathology as a major contributor of MS-related disability and the involvement of synaptic structures assumed a key role in the pathophysiology of the disease. Synaptic contacts are considered central units in the information flow, involved in synaptic transmission and plasticity, critical processes for the shaping and functioning of brain networks. During the course of MS, the immune system and its diffusible mediators interact with synaptic structures leading to changes in their structure and function, influencing brain network dynamics. The purpose of this review is to provide an overview of the existing literature on synaptic involvement during experimental and human MS, in order to understand the mechanisms by which synaptic failure eventually leads to brain networks alterations and contributes to disabling MS symptoms and disease progression.

## 1. Introduction

Multiple sclerosis (MS) is a chronic, inflammatory and degenerative disease of the central nervous system (CNS), a leading cause of neurological disability in young adults and a central public health issue in the world-wide population [1,2]. MS is considered to be an immune-mediated disease characterized by the occurrence of inflammatory lesions disseminated in time and space in the CNS, giving rise to a specific clinical course characterized by the onset of acute/subacute neurological deficits followed by partial or complete recovery. The repetitive activation of the immune system consisting of self-reactive immune cells that are able to cross the blood-brain barrier, the activation of local CNS immune cells and the release of pro-inflammatory mediators, characterize this early phase of the disease [1,3]. Over time, however, a large proportion of MS patients also experience chronic accumulation of neurological disability, involving different functional systems, from mobility to cognition [4]. Disease progression and disability accumulation in MS can be the result of complex mechanisms of networks disconnection due to the involvement of both gray and white matter. Focal gray matter lesions [5] and gray matter atrophy [6] were shown to interfere indeed with the function of both brain cortical structures and subcortical gray matter nuclei, while focal white matter lesions [7], axonal transection [8] and white matter atrophy [9] were seen to directly lead to circuit disconnection.

A further element that seems to critically contribute to the disruption of physiological brain dynamics is synaptic loss [10,11] and synaptic dysfunction.

Synaptopathy is known to be a common marker of neurodevelopmental disorders [12] and in the last decades growing attention has been directed towards the investigation of synaptic abnormalities, as key events also in the pathogenesis of neurodegenerative disease including Alzheimer’s disease [13,14,15], Parkinson’s disease [16,17,18] and Huntington’s disease [19], triggering neuronal loss through dying-back mechanisms or neurotransmitter imbalance in cortical and subcortical areas. A pathogenic role of synaptic dysfunction has been demonstrated not only in primary degenerative CNS disease, but also in neuro-inflammatory brain disorders such as MS [20,21]. Growing evidence suggests indeed a central role of the immune system and of soluble immune molecules in the regulation of synaptic transmission and plasticity in physiological conditions, with a central role in memory acquisition and consolidation [22,23,24]. Such intertwined relation between the immune and the nervous system might turn into a dysfunctional process during neuroinflammation.

Neuropathological studies in humans showed that widespread synaptic loss may accompany inflammatory white and gray matter lesions [10,11] and an inflammatory-driven failure of synaptic plastic properties has been described in experimental models of neuroinflammation [25]. Synapses are fundamental functional entities, expressing short- and long-term plastic changes, able to ensure learning processes, context-dependent input integration, multi-modal information processing and the ability to record, store and retrieve memory traces in cortical and subcortical brain networks [26,27]. Thus, their malfunction and loss might critically contribute to connection failure in the MS brain [11,20].

The aim of this review is to discuss recent findings on functional and structural synaptic involvement in both experimental and human MS to understand the different mechanisms by which synaptic failure might contribute to networks dysfunction, driving the onset and progression of MS-related disability.

## 2. Structural Synaptic Involvement in Multiple Sclerosis: Clues from Experimental Models of Neuroinflammation

Investigations performed in experimental models of neuroinflammation provided several inputs useful to decipher the pathogenic mechanisms underlying structural synaptic damage during MS. In 2003, Zhu and colleagues [28] showed extensive dendritic swelling in the spinal cord of rats affected by myelin basic protein (MBP)-induced experimental autoimmune encephalomyelitis (EAE) [28]. Dendritic spines abnormalities appeared to be dependent on the extent of local inflammatory cell infiltration, and were enhanced during the acute inflammatory phase of EAE [28]. The authors also showed a profound reduction in specific pre- and postsynaptic proteins (synaptophysin, synapsin I, and PSD-95) immunoreactivity, suggesting an alteration of synaptic structures in the lumbosacral spinal cord during the acute relapsing phase of EAE [28]. Such alteration was reversed during the recovery phase of the disease, reflecting the renewal of synaptic proteins and/or a plastic process of neosynaptogenesis [28]. Changes of synaptic density at the level of spinal cord motor nuclei was also assessed by Freira et al. in myelin oligodendrocyte glycoprotein MOG_35-45_-induced EAE [29]. Quantitative analysis of synaptic markers revealed a significant decrease of synaptic density at the disease peak, with recovery during the first remission phase, in parallel with the improvement of clinical signs. The ultrastructural analysis of synaptic terminals highlighted a progressive process of synaptic retraction during the course of the disease [29]. These data collectively suggest that major changes, occurring at the level of the synaptic structures during the peak phase of the disease, might contribute to the appearance of clinical signs in EAE and that chronic inflammation might impair synaptic recovery with time, resulting in cumulative synaptic damage [28].

Ziehn and colleagues confirmed the presence of gray matter damage during experimental MS, showing a significant decrease of the volume of the hippocampal CA1 region in MOG_35-45_ EAE mice compared with healthy matched controls [30]. Moreover, in the same brain region, where Schaffer collaterals afferents connect with pyramidal neuronal dendrites, an important reduction of synaptic density and presynaptic puncta (marked with immunostaining for Synapsin-1) was demonstrated [30]. Of note, during all the stages of the experimental disease, the authors detected abundant inflammation, which was primarily due to activated microglia and macrophages instead of infiltrating T and B lymphocytes [30].

Synaptic loss was also demonstrated in the somatosensory cortex of the EAE rat model induced with syngeneic spinal cord homogenate [31]. Specifically, a morphological analysis with specific dendritic markers at the level of the sensorimotor cortex revealed dendrites swelling and constrictions, similarly to what it was found at the level of spinal cord by Zhu and colleagues [28]. The authors further examined whether the dendritic tree and synaptic structures were damaged specifically in layer IV of sensory neurons, showing a reduction of spine density with a negative correlation between spine density and inflammatory infiltrates [31].

### 2.1. Microglia-Dependent Mechanisms Underlying Synaptic Involvement

In line with these findings, an emerging role in shaping synaptic networks both in the healthy CNS [32] and in pathological conditions [20,33,34,35] is attributed to soluble immune factors and microglial cells. For instance, Stevens and colleagues demonstrated that the classical complement cascade is required for synapse elimination during normal development of brain networks but could be aberrantly reactivated in the adult CNS after tissue injury or during the course of neurological diseases [32]. The complement- and microglial-dependent engulfment of synapses is a finely tuned mechanism, also depending on the release of soluble immune mediators [36,37]. In particular, Bialas and colleagues established a specific role for astrocyte-derived TGF-β in regulating neuronal expression of C1q and C3 elements of the complement system, with potential implications for synapses elimination in the diseased brain [36]. The tight cooperation between microglia and astrocytes in the process of synaptic pruning [36] could be altered during pathological inflammatory processes.

Interestingly, Hammond and colleagues recently investigated complement expression and contribution to synaptic damage during MOG_35–55_ EAE, with a focus on hippocampal area [38]. The authors analyzed C1q and C3 proteins and mRNA expression at the level of hippocampal region. They found a significant increase of the expression levels of both C1q and C3 complement components within the hippocampus of EAE mice compared to controls [38]. Moreover, the genetic deletion of C3 completely protected from EAE-induced synapse elimination, in parallel with an attenuated severity of EAE motor impairment, reduced microglial activation, and improved freezing and memory in contextual fear conditioning experiments [38]. In agreement with the previous study, Werneburg and colleagues identified synaptic loss at the level of the geniculate system in an EAE animal model at the onset of clinical symptoms (10–12 days post injection, dpi) [39]. They also observed significant increase in peripheral immune cell infiltration, without significant changes in myelin sheath and no changes in neuronal density, demonstrating that synapse loss can occur prior to neuronal degeneration and to myelin pathology but in the presence of local neuroinflammation [39]. Interestingly, the investigators found that the engulfment of presynaptic terminals within microglial lysosomes correlated with the increase in C1q and C3 complement factors expression in the retinogeniculate system, but only the complement factor C3 co-localized with presynaptic terminals [39].

### 2.2. Other Potential Mechanisms Underlying Synaptic Involvement

Synaptic loss during EAE has been demonstrated to occur also independently from microglial activation. Indeed, Yang and colleagues found that early alterations of synaptic connections in the primary somatosensory cortex correlated with increased peripheral TNF-α production, in the absence of increased production of this cytokine in CNS or microglial activation [40]. Specifically, using in vivo two-photon microscopy, they observed an increased turnover of both postsynaptic dendritic spines and presynaptic axonal boutons in the somatosensory cortex of pre-symptomatic MOG_35–55_ EAE mice (0–7 dpi). These early alterations of synaptic structural dynamics were observed in conjunction with increased peripheral production of TNF-α and the peripheral administration of anti-TNF- α agents was able to block the abnormal plasticity of dendritic spines and axonal boutons that were observed in pre-symptomatic EAE mice. These data suggest that TNF-α could play an important role in inflammation-mediated changes of synaptic connections [40], potentially acting through the activation of its neuronal receptor or by modulating the expression of other local pro-inflammatory cytokines [40]. These early synaptic connection instabilities seemed to be independent from focal white matter lesions, and were hypothesized to contribute to the behavioral changes occurring in EAE animals early in the disease course, before the onset of motor symptoms [40].

Lastly, mitochondrial dysfunction and oxidative stress may play and additional pathogenetic role in the damage of the neuro-axonal unit and synaptic terminals during neuroinflammation [41]. Indeed, axonal mitochondrial dysfunction represents an early event during the course of MS [42] and the inflammatory process associated with EAE enhance mitochondrial susceptibility to metabolic stressors [43]. In this scenario, it is possible to hypothesize a failure of the mitochondria located in the presynaptic terminals, which exhibit an inherent vulnerability to metabolic stressors [44] and play a key role in maintaining the physiological structure and function of synapses [45]. The production of reactive oxygen species (ROS) and free radicals by immune cells can represent a link between inflammation and neurodegeneration during MS [43,46,47,48], with the possible activation of signaling pathways leading to synaptic degeneration, as a proem to neuronal loss.

Overall, preclinical evidence shows that structural synaptic damage is a widespread feature of experimental MS, involving several CNS regions (hippocampus, spinal cord, optic nerve, geniculate system), suggesting ubiquitous CNS synaptic damage during neuroinflammation. An emerging role is attributed to soluble immune molecules, complement factors and microglial cells, giving rise to a complement-dependent model by which microglia engulf and eliminate synapses during EAE [38,39], revealing early neuro-immune pathways as possible therapeutic targets for the modulation of networks dynamics during MS.

## 3. From Structure to Function: Synaptic Transmission in Experimental MS

Accumulating evidence suggests that the immune system physiologically modulates synaptic transmission and neural networks functioning, by releasing soluble factors and promoting a continue crosstalk between neuronal elements and resident or infiltrating immune cells [49,50]. The presence of a chronic and unabated inflammatory process within the CNS may alter these delicate neuro-immune interactions: accordingly, a functional alteration of basal synaptic transmission, beyond structural damage, is thought to occur during MS.

### 3.1. Excitatory Glutamatergic Transmission

Studies performed in a MOG_35–55_ EAE model showed an abnormal glutamate-mediated excitatory neurotransmission in the nucleus striatum [51], the key input station of the basal ganglia circuit. The duration of spontaneous and miniature excitatory postsynaptic currents (sEPSCs and mEPSCs) was found to be increased in striatal medium spiny neurons (MSNs) of EAE mice, compared to control animals, both in the pre-motor (7–10 dpi) and acute clinical phase of the disease (20–30 dpi). This increase in glutamatergic transmission was associated with an altered trafficking and increased phosphorylation of the α-amino-3-hydroxy-5-methyl-4-isoxazolepropionic acid (AMPA) ionotropic receptor [51]. Indeed, the application of the AMPA receptor antagonist NBQX was able to reduce motor symptoms and dendritic spine loss in EAE animals [51]. Interestingly, the authors demonstrated that the incubation of control slices with activated microglia or TNF-α was able to mimic synaptic abnormalities observed during EAE, attributing a central role to both microglia and TNF-α in the modulation of the sensitivity of AMPA receptors to synaptically released glutamate.

Grasselli and colleagues provided evidence of a further molecular mechanism triggering enhanced glutamate release in the striatum of MOG_35–55_-induced EAE mice [52]. The authors demonstrated that an abnormal activation of *N*-methyl-d-aspartate (NMDA) receptors localized at the presynaptic site contributed to increased striatal sEPSC frequency during the acute EAE phase (20–30 dpi) [52]. Moreover, they showed that the in vivo pretreatment (1 week before immunization) of EAE mice with a selective antagonist of the NMDA receptor improved early EAE clinical symptoms and delayed the acute phase of the disease [52]. Conversely, the induction of EAE in mice with genetically enhanced NMDA receptor activity (in mutant mice lacking the d-aspartate oxidase) was characterized by opposite effects [52].

The mechanisms underlying altered glutamatergic transmission have also been characterized in other brain areas during EAE. Electrophysiological recordings from cerebellar Purkinje cells showed an increased duration of sEPSCs during the acute phase of MOG_35–55_ EAE (20–25 dpi), indicating that spontaneous glutamatergic transmission is altered also in this brain structure [53]. Interestingly, such synaptic alterations were found to be dependent on the loss of physiological functions of astrocytes. Astrocytes were long considered as passive players in the pathogenesis of MS, being responsible of glial scar formation [54]. However, astrocytes were found to actively participate in the initiation and progression of MS-related inflammatory process [55]. Astrocyte activation is triggered by soluble pro-inflammatory factors [56] and plays a key role in sustaining the recruitment of immune cells in the CNS [57]. In addition, the astrocytes may express a pro-inflammatory profile, generating oxidative stress and increasing the free radical content in the CNS microenvironment [58], with a concomitant loss of their physiological role as synaptic sensors [53,59]. Specifically, astrocytic glutamate re-uptaking is essential for the maintenance of a proper synaptic transmission and plasticity [53,58,59]. During EAE, it has been shown that the prominent downregulation of glutamate-aspartate transporter (GLAST), which is the main glutamate transporter expressed by Bergmann glia at Purkinje synapses, results in a defect of astroglial glutamate uptake from the synaptic cleft [53]. The expression of GLAST was inversely correlated with the cerebellar concentration of the pro-inflammatory cytokine interleukin-1β (IL-1β). Interestingly, the same research group also demonstrated that GLAST represents a target of miR-142-3p, a specific microRNA upregulated in the cerebrospinal fluid (CSF) of MS patients and in EAE cerebellum [59]. Collectively, the authors identified a regulatory IL-1β/miR-142-3p/GLAST axis able to trigger the enhancement of sEPSCs duration in minutes (slower decay phase and half-width of sEPSCs kinetics) [59]. Of note, the synaptic effects of IL-1β, both in terms of glutamatergic dysfunction and GLAST regulation, was absent in slices coming from miR-142 KO mice [59].

The hypothesis that EAE-related alterations of synaptic transmission rely on the release of pro-inflammatory soluble molecules during neuroinflammation is supported by several studies. In particular, Rossi et al. exposed, in vitro, murine brain slices to human CSF obtained from active and quiescent MS patients [60]. Interestingly, these authors found that the exposure of brain slices to CSF of MS patients with signs of active inflammatory brain lesions at magnetic resonance imaging (MRI) examinations was able to increase striatal sEPSC frequency and to induce glutamate-mediated neuronal swelling in vitro, through a mechanism dependent on IL-1β and increased AMPA receptor stimulation [60]. In particular, they identified, as critical molecular targets of IL-1β, the transient receptor potential vanilloid 1 channel (TRPV1) located at presynaptic glutamatergic terminals [60] and the CB1 receptors [61], which are known to orchestrate and regulate glutamate transmission.

### 3.2. Inhibitory GABAergic Transmission

The γ-aminobutyric acid (GABA) is the major inhibitory neurotransmitter in the CNS; accordingly, an important mechanism to counteract excitotoxicity in MS brain could be represented by an increase of the GABAergic transmission. Early reports showed in the spinal cord of EAE animals a reduction of both GABA and of its synthesizing enzyme, glutamic acid decarboxylase (GAD) [62] or of the GABA transporter 1 (GAT-1) [63], suggesting an alteration of GABAergic transmission during the course of the disease. Accordingly, it has been shown that, in the acute phase of MOG_35–55_ EAE (20–25 dpi), the frequency and amplitude of spontaneous inhibitory postsynaptic currents (sIPSC) in the nucleus striatum was reduced compared to controls [64,65]. It has been proposed that the alteration of GABAergic transmission could be triggered by the exposure to soluble inflammatory mediators, since the prolonged exposure of cultured neurons to a cocktail of pro-inflammatory cytokines (IL1-β, IFN-γ and TNF-α) markedly perturbated the GABAergic system mimicking the effects of a GABA-A receptor antagonist [64]. In line with these results, the same research group demonstrated a significant reduction of striatal sIPSCs amplitude in slices incubated with CSF coming from active MS patients [66]. The blockade of TNF-α signaling had no positive results but the supplementation of CSF from active patients with an IL-1β receptor antagonist completely prevented the reduction of striatal sIPSCs amplitude [66]. Interestingly, other works suggested that the IL-1β-induced GABAergic defect might be mediated by mechanisms similar to those influencing glutamatergic transmission. Indeed, it has been shown that the genetic or pharmacological inactivation of TRPV1 channels abolished the IL-1β-mediated effects on GABAergic synapses [67] and this may rely on the TRPV1-dependent modulation of the sensitivity of presynaptic endocannabinoid CB1 receptors controlling GABA release [68].

A possible IL-1β-dependent alteration of GABAergic transmission has been found also in other brain areas. Specifically, a strong reduction of the frequency of the sIPSCs was found also at the level of cerebellar Purkinje cells [69]. In particular, during the symptomatic phase (20- and 23-dpi) of the disease, the authors showed a strong reduction of the frequency of sIPSCs recorded from cerebellar Purkinje cells, together with a prominent microglia activation in the white matter and in the molecular layer of the cerebellum [69]. Additionally, in this brain area, the incubation of mice brain slices with IL-1β was able to mimic the electrophysiological alteration found in the cerebellum of EAE mice [69]. A reduced sIPSCs amplitude and larger inter-event interval were also found in the hippocampal region of MOG_35–55_ EAE mice, with GABAergic transmission abnormalities being mimicked by the exposure of control slices to IL-1β [70]. In the hippocampus, an opposite result was found by Kammel and colleagues [65] who demonstrated an enhancement of sIPSCs at 21–42 dpi. Interestingly, Kammel and colleagues also found an enhancement of hippocampal tonic neuronal inhibition in association with an increased surface expression of α5 subunit-containing GABA_A_ receptors [65] and hypothesized a role of altered GABAergic neurotransmission in hippocampus-dependent cognitive dysfunction in EAE and MS. Interestingly, other studies [71,72] showed that the pro-inflammatory cytokine IL-1β is able to induce a rapid increase in GABA_A_ receptor surface expression in hippocampal neurons [71]. This evidence suggests that inflammatory mediators, such as IL-1β, can affect cell excitability in the hippocampal region acting on GABA_A_ receptors.

Overall, these data support the notion that synaptic transmission is altered during EAE. Specifically, evidence suggests the presence of unbalanced synaptic glutamatergic and GABAergic transmission in different brain regions, probably driven by pro-inflammatory soluble molecules overexpressed in this pathological condition.

## 4. Synaptic Long-Term Storage and Network Modelling

One of the most interesting properties of the brain is the ability to retain memories and to shape neural networks on the basis of previous experience. The modulation of neuronal connection strength, modifying the structure of synapses in response to specific patterns of electrical activity inducing long-term synaptic changes, has been named long-term potentiation (LTP) and depression (LTD) [73,74]. These fascinating synaptic properties, able to model and refine neural networks by strengthening effective neuronal connections while weakening others, are considered as the putative neurobiological base for learning and memory processes [75,76]. Bidirectional plasticity is required to guarantee proper homeostatic functioning, allowing the compensatory weakening of neighboring synapses when a specific connection undergoes potentiation [77].

In MS, brain plastic abilities are involved in recovery and adaptive mechanisms associated with white and gray matter damage, preserving neural networks activity, and coping with the progressive reduction of neurological reserve that is associated with disability. Accordingly, the hypothesis that the long-term preservation of brain functional adaptive mechanisms might contribute to a more favorable course of the disease has been raised [78]. In this scenario, the evidence that the structural and functional synaptic plasticity might be impaired during MS [20,25,49,51] are highly relevant, since abnormal plasticity might counteract beneficial compensatory mechanisms.

**Figure 1:**
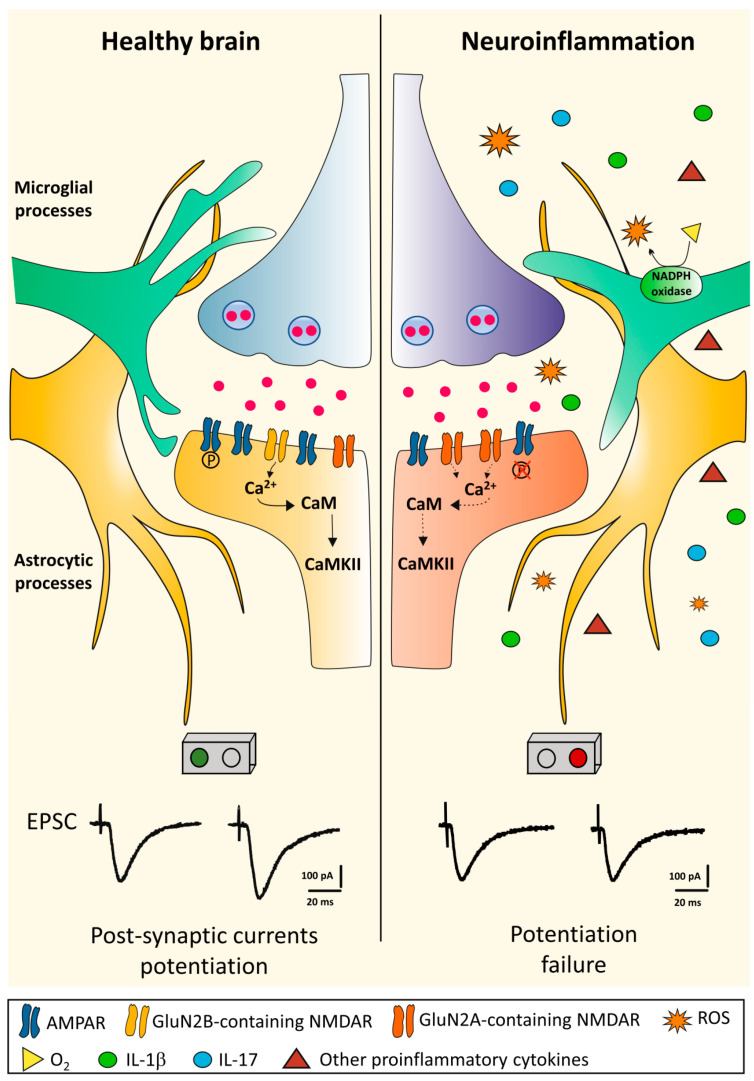
Long-term potentiation in the healthy brain and its disruption during neuroinflammation. In the healthy brain, glutamate released from the presynaptic terminal activates AMPA glutamate receptors (AMPAR) inducing membrane depolarization mediated by Na^+^-influx in the postsynaptic structure. Depolarization activates the NMDA glutamate receptors (NMDAR) allowing postsynaptic Ca^2+^ influx. The resulting increase in Ca^2+^ concentration within the dendritic spine activates a series of downstream pathways such as CaMKII. All these processes eventually lead to an increased current flow through the phosphorylation (P) of already expressed AMPARs and the addition of new AMPARs. In the late-phase of LTP, the signal cascade translocates to the nucleus inducing changes in gene expression and synthesis of new proteins, allowing structural synaptic remodeling. During neuroinflammation, the CNS microenvironment undergoes different changes. Microglial cells become activated by assuming an ameboid form and together with astrocytes and peripheral immune infiltrating cells release diffusible proinflammatory mediators leading to LTP failure [80,81]. The precise mechanisms underlying neuroinflammation-induced plastic failure are still under investigation, but a central role is attributed to the activation of innate immunity, increased expression of inflammatory cytokines and ROS production by neurotoxic enzymes, such as NADPH oxidase. These events are accompanied with a postsynaptic NMDA receptor rearrangement with reduced expression of the GluN2B subunit [79,80]. Abbreviations: AMPAR, α-amino-3-hydroxy-5-methyl-4-isoxazolepropionic acid receptor; CaMKII, Ca^2+^/calmodulin-dependent protein kinase II; LTP, long-term potentiation; NADPH oxidase, nicotinamide adenine dinucleotide phosphate oxidase; NMDAR, *N*-methyl-d-aspartate receptor; ROS, reactive oxygen species.

It has been proposed that inflammatory-related activation of both resident glial cells and infiltrating immune cells producing pro-inflammatory molecules could impair physiological synaptic plasticity. Synaptic plastic properties have been investigated in the CA1 hippocampal area in an experimental model of MS (chronic-relapsing experimental autoimmune encephalomyelitis; crEAE). Using this model, during the acute phase of the experimental disease (15–18 dpi) the LTP, a sustained form of potentiation, was found to be impaired. The impairment of hippocampal LTP in the acute phase of this EAE model was associated with an altered assembly of NMDA glutamate receptors and an increase in the hippocampal levels of the pro-inflammatory cytokine IL-1β. Moreover, immunohistochemical analysis showed a significant activation of microglial cells in the hippocampus of EAE animals [79]. Interestingly, synaptic plastic properties have been also investigated in the remission phase of this experimental model of the disease [80]. After the spontaneous resolution of EAE-associated motor deficits, CA1 hippocampal synapses still failed to fully express LTP, showing concomitant impairment of cognitive/behavioral performances, as demonstrated by an open field hole-board test [80]. Interestingly, these deficits were found to rely on the activation of hippocampal microglial cells, since the treatment with minocycline, a drug preventing microglial cells activation, was able to restore the CA1 LTP deficit and to rescue the cognitive abnormalities of EAE mice during the remission phase of the disease [80]. The microglia activation was accompanied by increased hippocampal levels of the pro-inflammatory cytokine IL-1β and by the over-expression of the ROS-generating enzyme nicotinamide adenine dinucleotide phosphate (NADPH) oxidase [80], suggesting a role of ROS in causing synaptic plasticity abnormalities (Figure 1).

In line with these results, another study supported a link between neuroinflammation, pathologic ROS production and impaired memory consolidation in MOG_35–55_ EAE [81]. Kim and colleagues showed changes both in cognitive abilities (assessed through the Morris water maze) and in hippocampal synaptic plasticity. Specifically, mice exhibited deficits in spatial motor learning even before the onset of EAE-associated motor deficits and the investigation of hippocampal LTP showed an impairment of these sustained form of potentiation at two different time-points after immunization (peak and milder disability) with respect to control animals [81]. These electrophysiological abnormalities were associated with hippocampal volume loss, documented at the same time points with MRI investigations [81].

Additional studies suggested that the disruption of synaptic plasticity in EAE might be time- and region-dependent. With regard to the timing of synaptic alterations, Novkovic and colleagues showed that CA1 hippocampal LTP was normal in the early phase of the MOG_35–55_ EAE model (14–19 dpi), but pathological alterations of LTP in this area and spatial memory deficits developed during the late phases of the experimental disease (40-45 dpi) [82].

On the other hand, with regard to the region-specificity of synaptic involvement, Prochnow and colleagues focused on the acute phase of MOG_35-45_ EAE mice (12–14 dpi), showing that LTP and LTD induction in CA1 hippocampal area was similar to controls [83]. Significant LTD changes in EAE animals, however, were demonstrated at the level of superior colliculus (SC) and cerebellum during the acute phase of the experimental disease [83]. More recently, Kammel and colleagues demonstrated that the induction of LTP was significantly reduced in hippocampal slices from MOG_35-55_ EAE mice both at early (16–29 dpi) and late time-points (40 dpi) of the experimental disease [65]. This impairment of LTP was found in association with an increase of tonic neuronal inhibition in the hippocampal region; however, the pharmacological reduction of tonic inhibition failed to restore LTP induction, suggesting a more complex scenario, also involving excitatory transmission in the pathogenesis of LTP impairment [79]. Overall, even if differences in the utilized experimental models or electrophysiological recording protocols may have led to partially conflicting results, preclinical studies collectively suggest that the neuroinflammatory process accompanying experimental MS is associated with altered synaptic plastic abilities, probably due to a detrimental role exerted by activated innate immune cells, soluble inflammatory molecules and ROS on synaptic function and postsynaptic receptors assembly. Moreover, the direct association between synaptic plasticity defects and altered cognitive performances, found in animal models, suggests that altered plasticity might result in impaired networks dynamics [80,81].

## 5. Synaptic Involvement in Human MS: Insights from Pathology

Several histopathological studies in humans confirm the presence of widespread damage of gray matter in MS brains, with a specific focus on synaptic loss [10,11]. A reduction of dendritic arborization and synaptic densities emerged, indeed, as an important feature of MS cortical and subcortical pathology, with the potential to compromise the functionality of these regions [84]. Several studies focused the attention on the hippocampal region [85,86]. Here, the lesions were characterized by a relative paucity of inflammatory cells, with activated microglia being the main inflammatory cell type [86]. Dutta and colleagues performed morphological and molecular analysis of hippocampi with and without demyelinating lesions from postmortem MS brains [10]. They used confocal microscopy to quantify the density of presynaptic terminals in specific hippocampal regions (CA1 and DG), demonstrating that loss of myelin is associated with a significant decrease in the number of synapses in MS brains. Moreover, the authors established that hippocampal demyelination leads to decreased expression of neuronal proteins involved in axonal transport, synaptic plasticity, glutamate homeostasis, memory/learning processes and neuronal survival [10]. These data suggest that demyelination is paralleled by relevant signs of synaptic damage during MS [10]. In 2016, Jurgens and colleagues used the Golgi-Cox impregnation technique to reconstruct the dendrites of cortical layers in order to better understand the microanatomy of gray matter pathology during MS. The investigators found a widespread loss of dendritic spines in the cortex of MS patients compared to controls. Interestingly, the reduction of spine density was equal in both demyelinated and normal-appearing areas of the cortex, suggesting that the widespread loss of synaptic connections occurs independently from white matter lesions [11]. The authors also quantified the density of cortical fibers that mostly represent afferent intra- and extra-cortical axons, demonstrating a significant reduction of cortical axon density only in the demyelinated cortex [11]. Together, these findings suggest that spine loss in the normal-appearing gray matter could represent a sign of primary synaptic damage in MS brains, not necessarily triggered by demyelination and/or axonal loss [11].

In a recent study, Werneburg and colleagues assessed synaptic connectivity at the level of the lateral geniculate nucleus of the thalamus in post-mortem tissues from MS and control patients. The authors found important decrease in retino-geniculate presynaptic terminals in MS patients compared to controls. Moreover, co-labelling microglia and macrophages, the investigators showed that the presynaptic terminals were engulfed within active microglial cells [39].

Overall, these data confirm the cardinal role of gray matter pathology during MS. In this scenario, synaptopathy emerges as an early fundamental event during the disease course (Table A1 in Appendix A), in part independent from demyelinating lesions.

## 6. CSF Biomarkers of Synaptic Integrity in Human MS

Compared to pathology investigations, CSF biomarkers offer the chance to in vivo investigate the pathophysiological mechanisms underlying MS and their relation to synaptic involvement and cognitive dysfunction. Although most robust evidence in MS has been produced on immunological markers, great attention has been recently directed towards neuronal biomarkers [87]. Specifically, axonal biomarkers, such as neurofilament light chain (NfL), are now close to becoming clinically useful [88]. Compared to axonal markers, synaptic markers are further back in the validation process. However, in the last decade, advances in mass spectrometry and immunoassays have allowed the identification of different synaptic proteins in biofluids [89]. Most of them, such as synaptotagmin-1 (Syt-1), synaptophysin, synaptosomal-associated protein 25 (SNAP-25), synaptic vesicle glycoprotein 2A (SV2A), α-synuclein (α-syn), and growth-associated protein 43 (GAP-43), are presynaptic proteins involved in synaptic vesicle assembly and neurotransmitters release. Other biomarkers consist of postsynaptic proteins, such as neurogranin and neuronal pentraxins (NPTX) [89]. Most of the evidence on these proteins as CSF biomarkers comes from studies on neurodegenerative diseases, such as AD, but preliminary results have been obtained also in MS. GAP-43 is a membrane-associated protein and a major component of the motile growth cones of elongating axons and immature synaptic terminals [89]. Of interest, it is mainly expressed in the hippocampus, entorhinal cortex, and neocortex of the adult brain [89]. Since GAP-43 is highly expressed during synaptogenesis and axonal outgrowth, it is considered as a marker of neuronal growth and synaptic regeneration [90]. Overall, its levels in the CSF seem not to be significantly different between early MS patients and healthy controls [91], while they are lower in progressive MS patients compared to controls [90]. These data suggest that, while in the earliest stages of the disease, the synaptogenesis is potentially preserved, in the progressive phases of the disease it could be impaired. In line with this, CSF GAP-43 has been shown to negatively correlate with age, disease duration and disability scores, with this latter association being significant independent of age in progressive MS patients [90]. Although a lower concentration of CSF GAP-43 can suggest a reduced potential of synaptogenesis, a transient increase of its CSF levels may still be an unspecific after-effect of CNS acute inflammatory injury. Like other neuronal biomarkers (e.g., NfL), CSF GAP-43, indeed, is higher in MS patients with a recent relapse and in those with gadolinium-enhancing lesions [90]. Additionally, a positive correlation between CSF GAP-43 and other inflammatory biomarkers (namely, CSF oligoclonal bands and CSF cells count) in MS has been reported [91]. Despite these similarities, no correlation between CSF markers of axonal damage (i.e., NfL) and GAP-43 has been found [91], thus suggesting that these two biomarkers reflect different pathophysiological mechanisms, with GAP-43 being characterized by a bidirectional behavior, where its CSF decrease may reflect a reduction in synaptogenesis potential and its CSF increase an ongoing neuronal and synaptic injury.

Unfortunately, no significant impact of disease-modifying drugs on CSF GAP-43 has been shown. Indeed, similar CSF GAP-43 concentrations have been found at baseline in patients without prior treatment and those on first line and second line treatments, thus suggesting that the anti-inflammatory effects of disease-modifying drugs do not influence synaptogenesis involving GAP-43.

Almost similar results have been obtained with CSF neurogranin, a protein concentrated in the dendritic and postsynaptic compartment of synaptic spines of neurons [89]. CSF neurogranin seems to be not significantly different between MS patients and controls [92], although in an independent cohort, lower values have been found in MS compared to healthy subjects [93]. The reason for this reduction is still unknown, but the presence of a lower density of dendrites in MS patients could be hypothesized. Within MS patients, CSF neurogranin has been found to be higher in those with gadolinium enhancing lesions compared to those without [92], confirming the evidence that acute inflammation in MS can be associated with synaptic damage and with subsequent transient increase in CSF neurogranin concentrations. However, as for GAP-43, neurogranin does not correlate with CSF NfL, which supports the hypothesis that its CSF changes reflect pathophysiological events different from axonal injury [92]. Moreover, for CSF neurogranin no significant impact of disease modifying drugs exposure has also been documented [92,93].

Finally, studies on the presynaptic protein α-syn, have demonstrated conflicting results, with both increased and reduced CSF concentrations being reported in MS patients compared to controls [94,95]. Such inconsistency may rely on different MS and control populations, as well as on different immunoassays and different pre-analytical variability sources, such as blood contamination.

In conclusion, CSF synaptic biomarkers may provide insights into the overall burden of structural synaptic derangement during MS, more than on its functional involvement. Evidence so far seems to show that CSF changes of these biomarkers may have a bimodal interpretation. While a reduced concentration of proteins such as GAP-43 and neurogranin appears to be an intrinsic feature of MS and likely represents the consequence of a reduced synaptogenesis potential and synaptic expression, on the other hand a transient increase of these proteins in the CSF could be a consequence of acute synaptic damage during acute focal CNS inflammation. The study of additional synaptic proteins that have been already investigated in the research of other neurodegenerative diseases, as well as the study of the correlation of these biomarkers with clinical and neuroradiological characteristics (e.g., gray matter volume and lesions), are crucial steps to better understand the contribution of these biomarkers in characterizing the synaptopathy taking place in MS.

## 7. Synaptic Involvement in MS: Consequences for Networks and Phenotypic Manifestations

As seen so far, an immune-mediated synaptopathy emerges from preclinical and clinical evidence as a key feature in MS. Different immunological pathways, as well as degenerative mechanisms, may contribute to the pathogenesis of MS-related functional and structural synaptic damage in different brain areas. The inflammatory process associated with MS may lead to an increased production of soluble immune mediators acting at synaptic sites in parallel with an alteration of microglial/astrocytic phenotype leading to synaptic transmission and plasticity dysfunction in cortical and subcortical networks [25,96].

**Figure 2:**
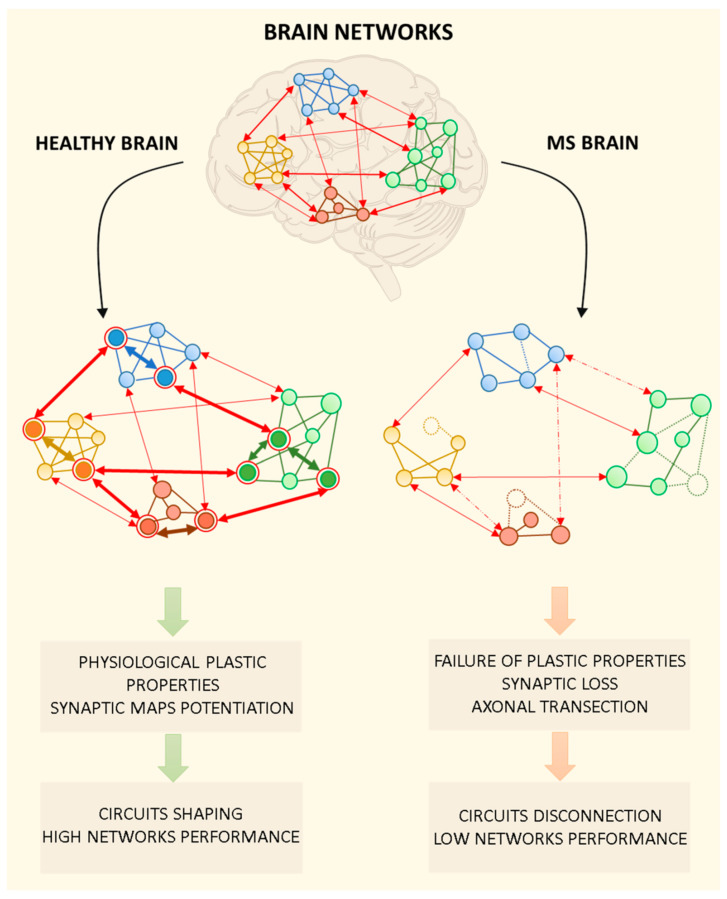
Brain networks in the healthy brain and during multiple sclerosis (MS). Brain networks are plastic structures in which multiple mechanisms are required to ensure physiological dynamics for multimodal information processing, context-dependent input integration, and learning mechanisms. In the figure, brain networks are represented as functionally interconnected brain regions. In particular, the graph is composed of round nodes representing specific neuronal ensembles belonging to different brain areas, intra-network connections and inter-networks projections. In the healthy brain, the physiological functioning of brain networks is made possible by synaptic plastic processes that allow to strengthen effective intra- and inter-network connections (bold arrows), while weakening others (not bold arrows). The shaping and refining of neuronal circuits lead to the selective activation of specific neuronal ensembles in the network (circled in red). Pathological activation of the immune system during MS is thought to affect synaptic structures and their functions. The MS brain is characterized by the loss of plastic mechanisms underlying the strengthening/weakening of intra- and inter-network connections. Moreover, the loss of neuronal connections due to axonal transection/damage (dashed arrows) and neuronal death (dashed nodes) contribute to network disconnection and low performance.

The clinical consequences attributable to synaptic damage are far from being fully understood, yet. Loss of physiological synaptic transmission and plasticity may be deeply involved in triggering brain networks dysfunction, cognitive impairment and disability during MS through different mechanisms (Figure 2). Withdrawal and rearrangements of synapses could explain the slowdown of information processing speed (IPS) in pivotal brain regions compromising the functionality of signal integration centers [97]. Alteration of IPS has been hypothesized to be a key characteristic of the MS brain and of MS-related cognitive impairment, potentially underlying the alterations in other cognitive domains according to the “relative consequence model” [27,98]. Interestingly, IPS impairment may rely on macroscopic and microscopic thalamic damage, since IPS performance was found to correlate with reduced thalamic volume and increased microstructural thalamic alterations assessed by diffusion tensor imaging and fractional anisotropy measures [26,99,100]. Basal ganglia and thalamus atrophy contribute independently to visual and auditory defects of IPS in MS patients [101] and several studies demonstrated, in the basal ganglia, reduced functional connectivity at the resting-state in MS patients [102], with a particular involvement of cortico-striatal motor loop [103]. Moreover, other reports have suggested that cerebellar involvement may be associated with impaired IPS [104]. Cortico-cerebellar loops have a role in attentional processes and atrophy of the posterior lobules of the cerebellum have been associated with alterations of IPS during MS [105,106]. As previously discussed, several preclinical studies supported the presence of striatal, thalamic and cerebellar synaptic dysfunction during pathological neuroinflammation. An immune-mediated alteration of basal synaptic transmission and synaptic plastic changes might significantly alter the efficiency of information processing in cortico-subcortical and cerebellar networks, acting as additional pathogenic factors for IPS impairment during MS.

The inability of synapses to change in response to specific stimuli and the loss of homeostatic cortical and subcortical networks activation through plastic phenomena may significantly alter the attentive, mnemonic and learning abilities of persons with MS [20]. Bidirectional plastic mechanisms are indeed also responsible for the ability to favor the processing of relevant information by reducing the interference produced by distracting events, thus increasing the signal-to-noise ratio in specific neural circuits; mechanisms believed to underlie the process of attention [107]. In addition, long-term synaptic plastic changes contribute in ensuring a proper input specificity and input divergence in neural networks, leading to the integration and storage of multimodal information during the process of conscious and unconscious learning. If the molecular mechanisms allowing simultaneous bidirectional plastic changes of synaptic transmission are impaired, or a balanced release of excitatory and inhibitory neurotransmitters is compromised by the occurrence of immune-mediated structural and functional synaptic damage, neuronal circuits may be unable to proper guarantee the mechanisms underlying attention, information storage and recalling. This could be critically important, considering that, during experimental MS, synaptic dysfunction has been described in brain structures exerting key cognitive roles, such as the hippocampus and striatum. Loss of hippocampal synaptic long-term plasticity has been linked to hippocampal-based visuo-spatial memory deficits in experimental models of MS [80,81]. The evidence of a correlation between synaptic plasticity deficits and hippocampal-dependent memory tasks is particularly important, since memory represents the second most common cognitive domain affected by network dysfunctions in MS [108].

Interestingly, what seems relevant in the pathogenesis of cognitive symptoms in people with MS is the initial level of the so-called “cognitive reserve” [109]. During a person’s lifetime, social interactions, intellectual enrichment (reading, learning, study carried out during the patient’s life) and experiences stimulating cognitive abilities, promote the formation and maturation of new synapses and neural circuits. It is therefore evident that the greater the cognitive reserve developed during life, the slower its decline will be along the course of the disease. [110]. In other words, the more active and stimulated the network of neuronal synapses through plastic processes, the greater the global number of synaptic connections linking brain key nodes. Cognitive-motor stimulation through environmental strategies [111,112] and exercise [113,114,115] also showed beneficial effect during the course of the experimental disease, indeed, animals exposed to enriched environments or those undergoing physical exercise had a delayed and milder EAE course, with reduced axonal loss [114], microglial reactivity, and demyelination in the spinal cord [112,113,114] compared to inactive EAE mice. Beyond the possible immune-modulating effects of physical activity, exercised EAE presented increased synaptic density of motor neurons [113] and recovered spine density in striatal neurons [115], suggesting a beneficial effect on synaptic function/structure. Overall, physical and cognitive stimulation may enhance the resilience and the recovery abilities of brain networks, in parallel with a modulation of the inflammatory process associated with EAE. The preservation of synapse functioning and neuroplastic mechanisms through adequate rehabilitation strategies may represent an effective therapeutic goal to counteract the detrimental consequences of immune-mediated synaptic dysfunction in people with MS [116].

## 8. Conclusions and Future Perspectives

Gray matter pathology, and in particular synaptopathy, emerges as an early and central feature in the course of MS. Both preclinical and clinical studies support the hypothesis of a pathogenic role of synaptic loss and malfunction in the pathogenesis of network disconnection and loss of functional reserve that eventually leads to irreversible disability. Although some peculiar pathogenic mechanisms of MS-related synaptopathy have been already elucidated, most of them still need to be unraveled.

Currently available and authorized therapies for MS prevent the formation of new inflammatory lesions in both the white and gray matter by exerting an immune-modulating effect. The potential benefits on neuronal and synaptic elements are thus related to the modulation of the CNS inflammatory milieu, but the use of novel targeted strategies aimed at safeguarding synaptic structure and function could be crucial for the recovery and preservation of brain networks function during the course of MS. Thereby, further preclinical efforts should be directed towards the identification of new molecular mechanisms underlying MS-related synaptic damage and new synaptic therapeutic targets, with the aim to boost adaptive plastic phenomena and restore networks dynamics in the MS brain. The discovery of new synapto-specific body fluid biomarkers could allow the in vivo investigation of MS pathophysiology, with the aim to obtain possible tools for the early detection of synaptopathy during MS and its relationship with specific disease features (such as cognitive impairment, disease progression, response to therapy). Moreover, the availability of reliable biomarkers may be useful to assess in the clinical setting the potential beneficial effects of the future synapto-centric therapeutic strategies.

## Appendix A

**Table A1:** Summary of main findings from human and experimental MS.

Main Findings from Human and Experimental MS	Quoted Literature
Altered expression of synaptic proteins/receptors	[10,28,30,38,39,51,65,79]
Synaptic/dendritic loss	[10,11,28,29,30,31,38,39,84,86,115]
Altered excitatory glutamatergic transmission	[51,52,53,59,60,61]
Altered inhibitory GABAergic transmission	[64,65,66,67,68,69,70]
Synaptic plasticity dysfunction	[65,79,80,81,82,83]
